# Supplementary figures and images for: Feeding growing button mushrooms: The role of substrate mycelium to feed the first two flushes
Source: PLoS One. 2022 Jul 26;17(7):e0270633. doi: 10.1371/journal.pone.0270633 (PMC9321441; doi:10.1371/journal.pone.0270633)

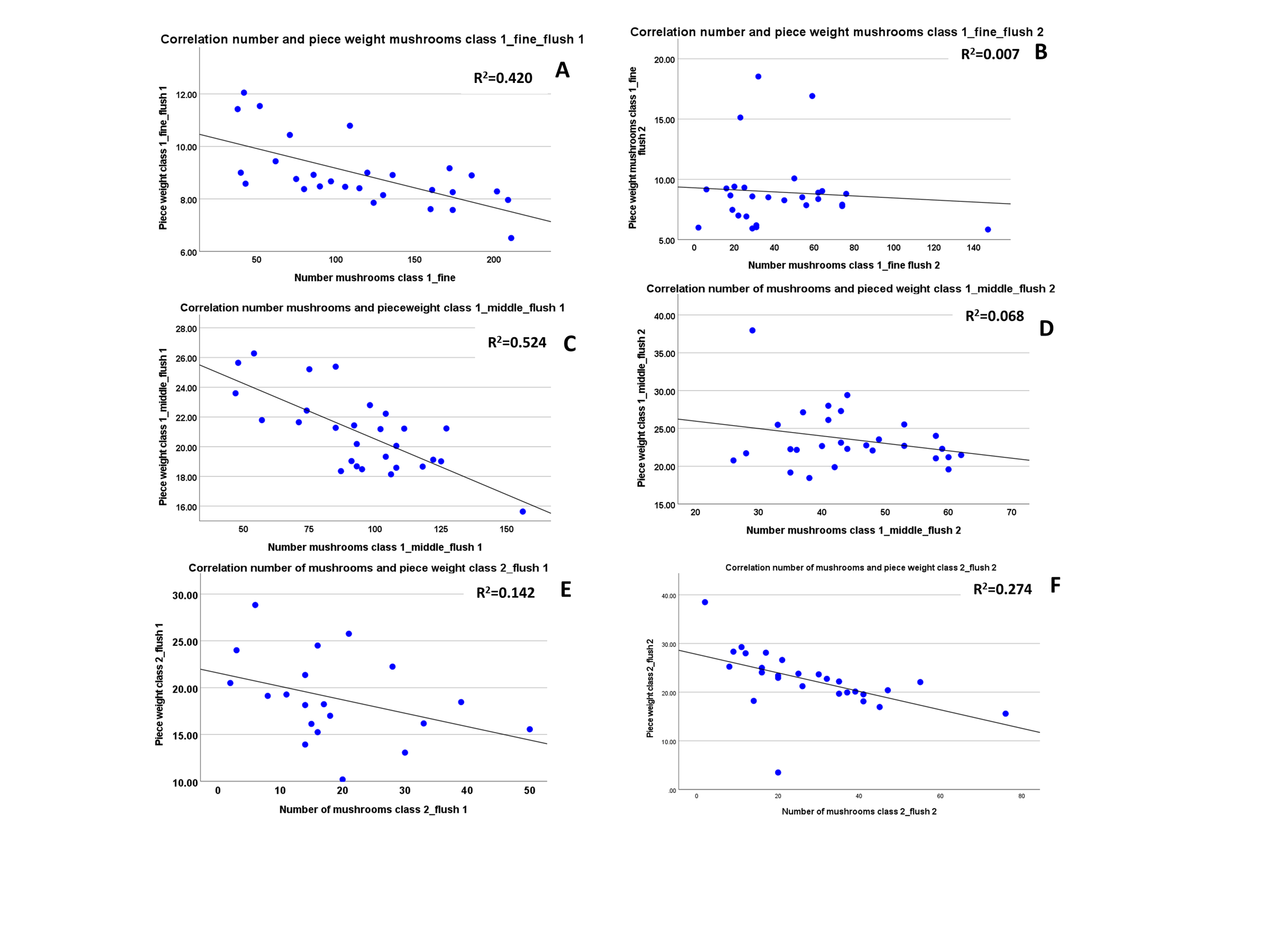

Supplement: S1 Fig — A&C: A negative correlation in flush one between piece weight and number of mushrooms for quality Class I fine and middle (Pearson’s Correlation r = - 0.648 and– 0.724, respectively; p<0.001), while such correlation is absent in flush two (B&D). E&F: For Class II mushrooms there is a moderate (flush one, E) and stronger (flush two, F) negative correlation between piece weight and number of mushrooms (Pearson’s Correlation r = -0.300 and– 0.523, respectively). (TIF) [file pone.0270633.s001.tif]

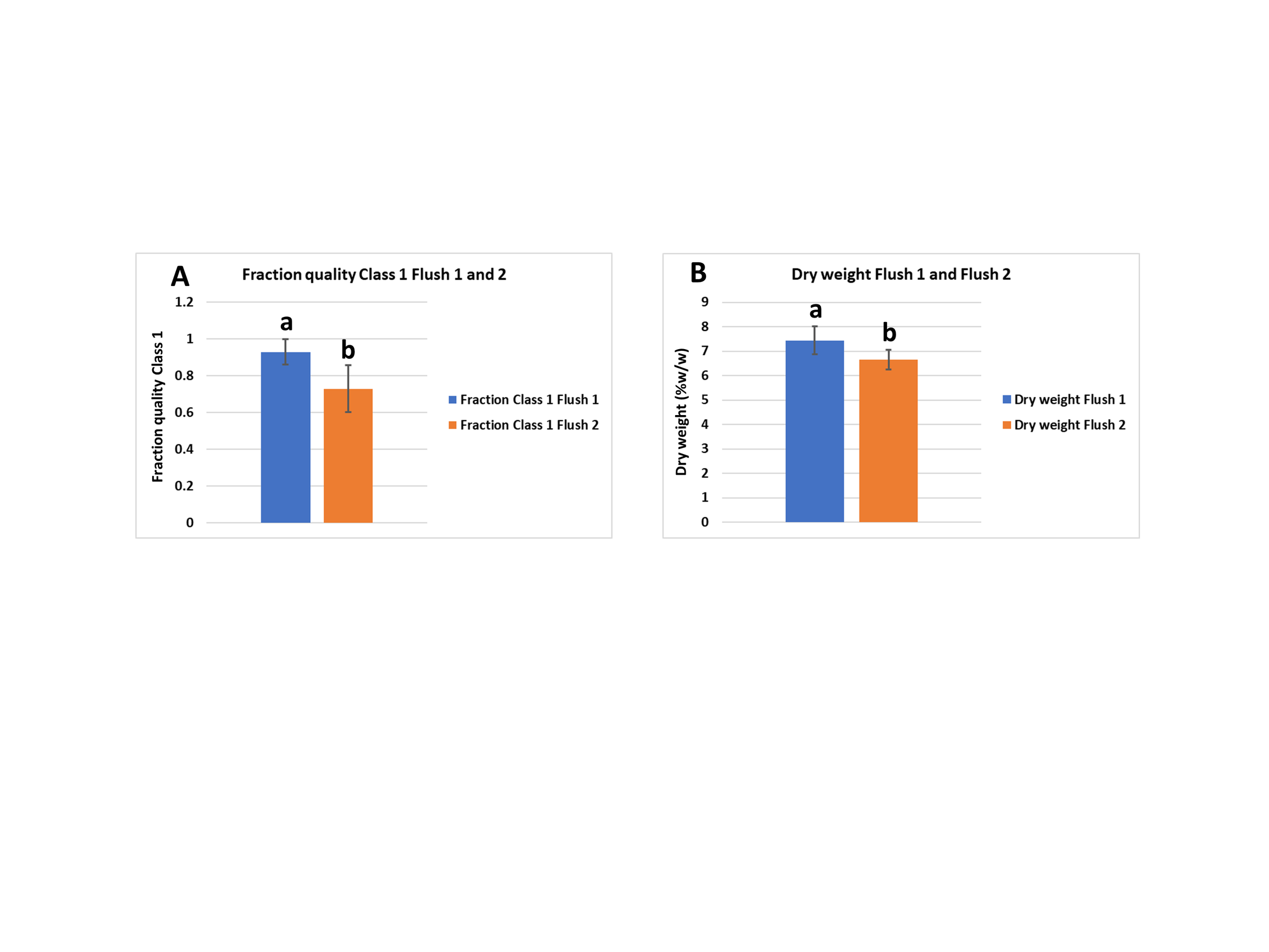

Supplement: S2 Fig — A: Significant difference in quality of mushrooms in flush one and flush two (p<0.001). B: Significant difference in dry weigh of mushrooms in flush one and flush two (p<0.001). (TIF) [file pone.0270633.s002.tif]

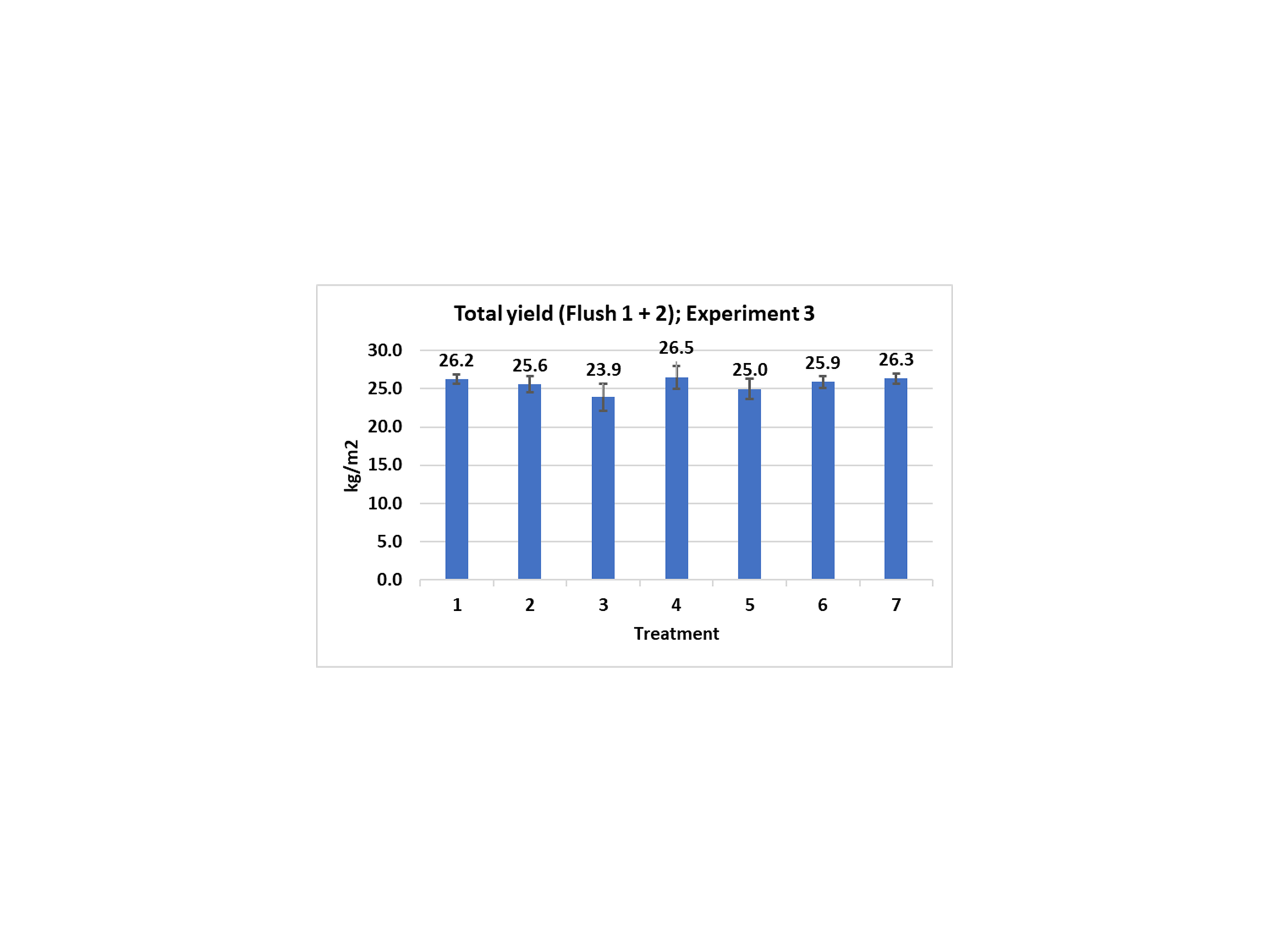

Supplement: S3 Fig — The short daily interruptions of contact between the casing soil and all or part of the substrate did not lead significant differences in te yield after two flushes. The error bars represent 2 x the standard deviation. (TIF) [file pone.0270633.s003.tif]

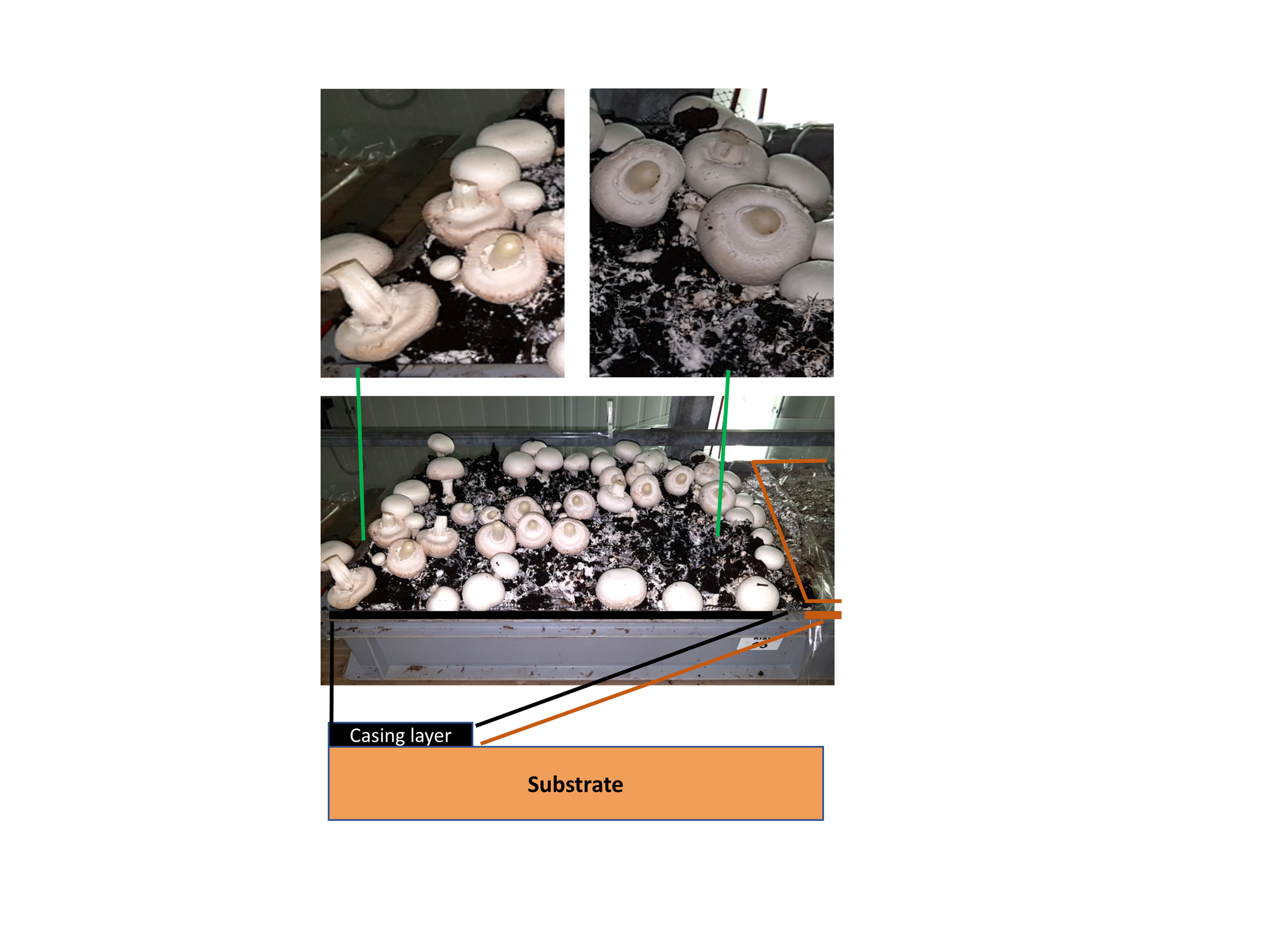

Supplement: S4 Fig — The bottom of the figure shows a schematic diagram of a cultivation tray with a sidewise extension of the substrate. The lower photograph shows the mushroom bed at the time of picking of flush one. The photographic enlargements of the top left and right show that mushrooms close to the substrate extension (left) have a better quality (less stretched velum) than the mushrooms at the opposite site (right). (TIF) [file pone.0270633.s004.tif]
