# Supplementary material for: Feeding growing button mushrooms: The role of substrate mycelium to feed the first two flushes
Source: PLoS One. 2022 Jul 26;17(7):e0270633. doi: 10.1371/journal.pone.0270633 (PMC9321441; doi:10.1371/journal.pone.0270633)
Supplement: S1 Data — (DOCX) [file pone.0270633.s006.docx]

**Raw data deposit: DOI 10.4121/19285106**
