# Supplementary material for: Feeding growing button mushrooms: The role of substrate mycelium to feed the first two flushes
Source: PLoS One. 2022 Jul 26;17(7):e0270633. doi: 10.1371/journal.pone.0270633 (PMC9321441; doi:10.1371/journal.pone.0270633)
Supplement: Attachment — Submitted filename: Rebuttal letter PLos One_Feeding growing mushrooms.docx [file pone.0270633.s008.docx]

Additional Editor Comments:

The manuscript is technically sound and will be suitable for readers working on mushrooms. The manuscript title may be changed for better understanding for readers. Moreover a spell check and English correction is required for entire manuscript.

The manuscript has been spell checked and a few miss spellings have been corrected.

Reviewer #1: The authors have planned the experiment very aptly to answer the questions on substrate degradation effect on the quality and quantity of the mushrooms. This research results have implications for both academicians and commercial mushroom growers. The authors have tried to support the study results with suitable literature review.

Thank you for your positive comments.
Line 41: cropping chambers instead of tunnels.
Phase II substrate is made in Europe in tunnels and not in cropping chambers.

Line 51: Whether the transport cost (mentioned in line 48) are same or different. Clarify.
Since >90% of the transport costs are labor and fuel, all types of transport costs are similar. I think that a clarification is not needed.

Line 63: “..filling phase III substrate...”. Alternate words can be used to convey its state like in terms of number of days from particular event or activity.
We are not sure what the reviewer intends to say. If the question is to specify days between the different events (from filling to end second flush), than these are indicated in, for example, figure 5.

Lines 65-66: “We also interfered with the system in three different ways”. Additional information desired.
The information on the interferences are given in the next sentences.

Line 135: “..the grids were shortly lifted..”. Approximately for what duration?
They were lifted and immediately placed back. It is irrelevant if the duration is 10 seconds or 1 minute, since the effect is the interruption of mycelial contact and the restoration time of mycelial contact is not dependent on the length of time were contact is broken.

Line 148: “...the same volume of substrate...”. Is it quantity?
“Volume” replaced with “weight”.

Line 207: “..in all layers but especially in the upper layer.” Replace “especially” with “more”.
Replacement made.

Line 211-212: “..,and now also degradation of the middle..”. Replace with clear stage of cropping as this indicates the observation is taken before concluding the experiment.
We are not sure what the reviewer intend to say here. The stage of cropping is the time in which flush one develops, as states in the beginning of the sentence. The “observations” are conclusions after samples have been analyzed after finishing of the crop.

Lines 387-388: “Mushrooms in flush two also clearly have a lower dry weight than mushrooms in flush one (flush one: 7.43 (% w/w); flush two: 6.65 (% w/w); p<0.001; Fig. S2B).” Is it possible to add possible reasons for the observation.
We have no clear explanation for the lower dry weight of mushrooms in the second flush. It might be that the balance between nutrients and water during the outgrow of the second flush tilts to the site of water. But that is highly speculative and, therefore, not mentioned in the discussion.

Line 408: “...~~height~~..”.
We discriminate here the placement of extra substrate as a sidewise extension or an increase of substrate depth, thus a placement directly under neat the casing. “height” is therefore appropriate.

Line 575: “Less reduced...”.
“Less substrate reduces..”

Reviewer #2: This is an interesting study and the manuscript was well drafted. Following are the few comments from my end
1. In view of the results mentioned in experiment -4 (from 297-299 lines), what should be the ideal depth of the substrate for the commercial production of Agaricus bisporus mushroom with the existing compost formulations?
2. It will be useful to the other co-workers, if the authors mention the compost and casing formulations used in the present study.

Thank you for your positive comment. As for your point 1: It is not easy to say what the best depth is for the present substrate formulation. The range (as stated in the manuscript) is what is used in Europe but each grower might use its own depth based on experience. It also depends on his ability to control the climate (and this the substrate temperature), when filling >100 kg/m^2^. The most growers use filling weight between 90 and 95 kg/m^2^. As for your point 2: I have added a reference in the M&M section of the manuscript (Jurak et al. 2015) which describes in detail the CNC substrate formulation.

Reviewer #3: The manuscript is technically sound and adequate data using different field and lab based approaches has been generated, analysed and properly inferred.

Thank you for our positive comments. And especially thank you for the thorough reviewing this manuscript.

The paper essentially deals with different facets of nutrient mobilization in button mushroom from different layers of substrate from casing to end of second flush. The title of the paper "Feeding growing button mushrooms. The role of substrate mycelium to feed the two first flushes." needs little correction and it can be Feeding growing button mushrooms: The role of substrate mycelium to feed the first two flushes.

The title has been changed.

There are minor corrections in the text as detailed below. At places there are unfilled dots to indicate that data is required here. These spaces may please be filled with appropriate data. Comments are mainly explanation for the comment and may be used for incorporating corrections wherever found relevant.

Line # : 2. Replace: two first flushes
With: first two flushes
Comments: The study deals with nutrient uptake in button mushroom from different layers from casing up to flush 2. The title can be more explicit.
Change have been made.


Line #: 17-18
Replace:1/3
With:1/3^rd^
Change have been made.

Line #: 28
Replace:20-50
With:20
Comments: In the absence of data at 30 and 40 cm, it is difficult to specify the exact distance
From the available data one can indeed not conclude what the exact distance will be. One can thus also not conclude that this distance is 20 cm. We have, therefore, changed the text into “..somewhere between 20-50 cm..”.

Line #: 46
Replace: matter by 16%, equivalent to 22% of the organic matter (2).
With: matter by 16%, equivalent to 22% of the organic matter (2) when compost is prepared using wheat straw ..%, chicken manure….%, ……gypsum…%.
Comments: This value of 22% is a function of the amount of gypsum used and mineral content of the straw or other substrate used and may be valid when ash content is around 27-28%. In many parts of the world paddy straw and other substrates are used and the quantity of gypsum also varies. Hence it is apt to give the exact formulation.
It is indeed true that the percentage of organic matter depends on the formulation of the substrate. This formulation is, however, fairly constant in European countries. The (small) variations made are a reaction on the quality of the raw materials received. These details are difficult to get from the companies. As explained in the introduction and M&M, we restrict our conclusions on European substrate formulations and used CNC substrate only. The formulation of this CNC substrate is defined in the reference given in the relevant M&M section.

Line #: 62
Replace: In order to further our understanding…
With: To further our understanding…
Comments: Use of prefixes like In order to, in case of in stead of just To or In is a personal choice and is not needed at may places
“In order..” has been deleted.

Line #: 94
Replace: used.
With: used. The bulk density of the compost after Phase II and its N content was…… and ……
Comments: Bulk density of compost, its nutrient status and watering schedule can impact the nutrient uptake/utilization from different layers. Hence conclusions may be only broadly applicable to different compost formulations. It is thus apt to specify the parameters of the compost used and watering schedule from casing to end of second flush. It may be added at appropriate places.
As stated above, substrate with a fixed substrate formulation was used in all experiments. Watering scheme of the casing soil is added in M&M.

Line #: 94
Replace: kg/ton
With: kg/ tonne
Comments: This may me corrected as per the pattern of Journal. At times ton and tonne are slightly different. Ton = 2240 pound whereas tonne is 1000 kg. (1000 kg =2204.6 pound)
Correction if agreed may be made throughout the paper at other places as well
Ton replaced with tonne throughout the manuscript.

Line #: below 97
Replace: 12
With: 4+8
Comments: In Table 1 Experiment 3 we have shown N as 12 where as it appears that we have 4 trays with no mesh and 8 trays with mesh between casing and compost (4 to be disturbed for 5 days and other 4 to be disturbed for 10 days). It is better if we write 4+8 at level 0 instead of 12.
Replacement done in Table 1.

Line #: 103
Replace: Trays with a surface area of 0.2 m2 were….
With: Trays with a surface area of 0.2 m2 (inside dimensions …..cm x ……cm) were….
Comments: We may give length x breadth. It is more relevant in Expt 3
Inside dimensions of the tray added in M&M.

Line #: 121
Replace: (equivalent to 82, 67 and 52 kg substrate/m2).
With: (equivalent to 82, 67 and 52 kg substrate/m2 and depth of …, …, and …. cm).
Comments: It will be apt if height is also mentioned. Or bulk density of compost is mentioned at any appropriate place
Filling height substrate id added in cm. Bulk density was not measured.


Line #: 146
Replace: same width as trays
With: same width (…..cm) as trays
Comments: Pl specify width
Width in cm added.

Line #: 147
Replace: but with different extended lengths and different depths
With: but with different extended lengths and different depths (……………….).
Comments: Pl specify the length at different depths
These are defined in Figure 2B.

Line #: 184-185
Replace: Samples were taken at 20, 50, 80 and 110 cm from the edge of the non-cased part.
With: Samples were taken from non-cased part at a distance of 20, 50, 80 and 100 cm from the edge of the cased area
Comments: Though it is understood what is meant, but non-cased part has three free edges. So it is better to correct the sentence. In abstract it has been put properly
Replacement made.

Line #: 186
Replace: 600 Erlenmeyer beaker
With: 600 Erlenmeyer flask
Comments: Normally Erlenmeyer is credited with flask and not beaker.
Replaced with Duran beaker. No Erlenmeyer flasks were used.

Line #: 188
Replace: 10.000
With: 10,000
Comments: I think common problem when we use different languages.
Correction made.

Line #: 217
Replace: is 20% ± 3,
With: is 20±3% or 20%±3 or 20%±3%
Comments: Please use which ever one is correct as per your data
20±3% etc. is used throughout the manuscript

Line #: 224
Replace: mushrooms per substrate depth in two flushes
With: mushrooms in three substrate depths in two flushes
Correction made

Line #: 226
Replace: p=0.422,, see
With: p=0.422, see
Comments: delete ,
Deletion made.

Line #: 229
Replace: We see also a in reduction of quality
With: We see also a reduction in quality
Change made.

Line #: 257
Replace: When looking at the Yields expressed per ton substrate, considering only the
With: ??
Comments: Incomplete line. Can be deleted or merged with the previous one
Incomplete sentence deleted.

Line #: 264
Replace: significant difference in yield flush two between
With: significant difference in yield of flush two betweenC
Change made.

Line #: 265
Replace: no significant differences yield flush 2 between 0 and 5
With: no significant differences in yield of flush 2 between 0 and 5
Change made

Line #: 344
Replace: contribution to flush two.
With: contribution to flush two. Thus there is gradual utilization from top layers to lower layers with advancement of flushes
Comments: Had we divided substrate into two layers only, then result possibly could have been:
During first flush top half layer is used, during period in-between two flushes nutrient from both layers are used and during 2nd flush material from the lower layer is mainly used. As we have tested only one system of 3 layers and not compared it with 2 or 4 layered system, hence, the conclusion needs to be balanced
Suggested sentence added.

Line #: 348
Replace: content in the tree layers
With: content in the three layers
Change made

Line #: 372
Replace: between te number
With: between the number
Change made

Line #: 389
Replace: ). There is no clear correlation between the number of mushrooms and their quality.
With: ). There is no clear correlation between the number of mushrooms and their quality up to yield level of ….ton/m2.
Comments: Overgeneralization. This is valid only at lower ranges. When the number of mushroom may increase beyond a level, these may affect quality
The sentence itself is indeed a overgeneralisation, but is has been made in reference to figure 4 where only three filling depths are displayed. But for clarity we added “..in the range of substrate depths used here”.

About Figures
Comments made are only suggestions and the ones deemed fit may be considered.

Fig 1 is a diagrammatic representation of different stages. To be more realistic we can show number of pinheads more than the mushrooms and number of mushrooms more in flush one than in flush two. However, pl see that it does not disturb the sampling arrows at the base.
Figure 1 has been modified as suggested.

Fig 2. Experiment 1. Premise seems to be that inserting mesh has no effect on fruiting. It would have been better to have one control for each mesh position to verify this premise. Alternatively, all the 28 bags could have three meshes at levels shown in Fig. 1. In that case, Tr 1 (Control): No mesh disturbed in four bags, Tr 2: top mesh disturbed for 5 days in four bags, Tr 3: top mesh disturbed for 10 days in four bags, Tr 4: middle mesh disturbed for 5 days in 4 bags, Tr 5: ….so on.
Please specify mesh/inch, wire dia, percent open area of mesh or other relevant specifications of the mesh used. (% open area = [1-(MxD)]^2 x 100 where M is mesh count or no. of wires per inch, and
D is diameter of wire in inches)
An extra treatment for each grid depth with a non-disturbed grid would indeed be the perfect control. We assume, however, that the inserted grid had no effect on the performance of the crop since the grid used had large mesh width (square mesh width of 3.6 cm and a wire thickness of 3 mm). We added in M&M: “The grids used in all experiments had a square mesh width of 3.6 cm and a wire thickness of 3 mm.”

Fig 3 Moisture content is same in all three layers in the beginning as Phase III compost is used which gets homogenized at the time of filling. Do we expect the same scenario of phase II compost is used in trays as is the trend in many countries?
That might differ since the physical conditions in a substrate layer after colonization might different from top to bottom. When filling with phase III substrate this putative inhomogeneity is avoided since phase III substrate is mixed well before filling.

Pl specify what error bars represent (±1 SD)
“Error bare represent ±1 SD.” Added in legend of Fig 3.
In Fig 3C, increase in dry weight in lower layer at end of flush 2 needs some explanation, or it is just a non significant deviation.
It seems indeed to be a non-significant deviation.

Here high moisture content in the top and bottom layer and low in middle layer at pinning in Fig 3 B seems to be the outcome of way we water the crop. It will be apt if we can add information about water sprayed/ day/m2 and how many times/day on different days from casing till end of flush 2.
Watering scheme has been added in M&M section.

Fig 4A. Title needs to be corrected to Yield (kg/m2; 2 flushes… .
Apparently increase in yield/m2 is valid only up to a limit after which it may plateau or even start decreasing due to excess heat or carbon dioxide production. Any data from other publications if available may be included in discussion.
Title corrected. Old data are available for higher filling weight and additional flushes, but since the substrate formulation and cultivation practices are so different to the present ones, a fair comparison is not reliable.

In Fig 4B yield kg/ton of substrate is an important aspect and the question commonly debated by growers that how much compost can be filled. This obviously depends on the cost benefit ratio as where the profits are higher, then even slightly less increase will be useful to the grower to get more mushrooms and hence more income per growing room. At the same time filling less compost not only decreases yield per room, but also affects the quality as discussed in the paper. Thus, it may be possible to define only the minimum filling depth. It will be apt if the same is made part of the conclusion and discussion.
It is difficult to define such minimum in general. In some countries, quality will be the most important factor defining the price of mushrooms, whereas in other countries the kilograms are the major factor for the economic profit for a crop. A general remark on this issue is made at the beginning of the ”The limits of the present system” of the Discussion paragraph.

Fig 5. It may be better to specify value at top of each bar (may be in vertical alignment mode). Absence of error bars suggests that it is sum total of all replicates. This will help in better understanding of the conclusion mentioned in the text that there is only shift in yield from flush 1 to 2 but overall yield of 2 flushes remains more or less same.
Error bars have been added in Figure 5. The translation of a date into days from the first picking day have not been done correctly. In the corrected data, the X-axis scale in Figure 5A is altered compared to the original figures leading to a delay in the last part of the second flush. In the appropriate section of the Result paragraph we have added :’’...and the extension of the duration of flush two.”
We have added a figure in the Supplemental data showing the total yield in a bar graph with error bars and the total yields as data labels above each bar.


Fig 6B Secondary axis may be corrected as: # of mushroom in flush 1/m2
Correction made. The R^2^ was missing in Figure 6B and has been added.

Fig 6C Primary axis may be corrected as : Yield Flush 2 (kg/m2)
Secondary axis may be corrected as: # of mushroom in flush 2/m2
Axis has been corrected.

Fig 9 has been titled as experiment 1. Instead of this in caption we can write that samples taken from Experiment 1
Caption has been removed and in the legends we added that the analyzed samples were taken from Experiment 1.

Fig 10: Secondary axis: RPKM can be expanded/mentioned in text
Added to legend of Figure 10: “ Expression of genes is expressed in RPKM (Fragments Per Kilobase Million).”

Fig 11: Looking at the dispersion of points, it will be apt to give R2 values for each graph
R2 values added.

S2 Correct sec axis …………. Class (instead of Clas)
S2B Correct secondary axis as …Dry weight (instead of Fry …)
Corrections made.

S4: Experiment 2: Normally SD values below a sample size are of less relevance and at the max indicate only degree of dispersion in data. In this experiment SD of 2 values has been calculated. Further, output up to 3 decimal is more than enough.
Total SD for each substrate kg/m2 seems to have been cultivated by just adding the basic data of all the three groups of supplementation. To compute the pooled SD from several groups, we normally calculate the difference between each value and its group mean, square those differences, add them all up (for all groups), and divide by the number of df, which equals the total sample size minus the number of groups. Its square root is the pooled SD. For example, for lines 19-21 we can pool SD =0.914011 (instead of 0.94073). Similarly pooled SD for 67 kg is 0.78 instead of 0.73 and for 82 kg it is 1.34 instead of 1.58. The differences are however miniscule and the values calculated have been used to only indicate the dispersion using error bars in bar or line diagrams. Due to low N (sample size), the df in different sources is low and it normally results in reduced efficiency of the test. In the present study, which is indoor study, it seems fairly OK to use less number of replicates considering the limited space available for experiments and uniformity of environment and other parameters during experimentation. This may be specified at any suitable place that the environment where experiments were cultivated were highly uniform and hence data from the limited trays was considered adequate.
For Experiment 2, there are indeed only duplicates for each supplementation level at each substrate depth. I have tried to recalculate the pooled SD according to the reviewers instruction, but cannot reproduce the numbers generated by the reviewer. We have used the standard one-way Anova in SPSS and consider this as appropriate for the statistics in Experiment 2.
I have added to the relevant M&M section that experiments were done in rooms with a good climate control.

We have also made a change in lines 621-622. “A production of ca. 15 kg mushrooms/m^2^, the former needs 27-31 kg substrate/m^2^ and the latter 85-110 kg substrate/m^2^.”
The calculation is incorrect and 15 kg/m2 for a first flush is also too low. The sentence has been changed into: “A production of ca. 18 kg mushrooms/m^2^, the former needs 21-28 kg substrate/m^2^ and the latter 85-110 kg substrate/m^2^.”
